# Comparative Immunogenicity of a High-Dose Hepatitis B Virus (HBV) Vaccine with Rapid Immunization vs. Standard Schedule in HBV Vaccine—Naïve Adults Aged 25–55 in China

**DOI:** 10.3390/vaccines12080923

**Published:** 2024-08-16

**Authors:** Qian Qiu, Huai Wang, Xiuying Liu, Xinghuo Pang, Wei Zhang

**Affiliations:** Beijing Center for Disease Prevention and Control, Beijing 100013, China; qiuq@bjcdc.org (Q.Q.);

**Keywords:** two-dose vaccination, high dosage, hepatitis B vaccine, immunization, adults

## Abstract

The 20 μg (0–1–6 month) hepatitis B virus (HBV) vaccination is widely recommended for HBV vaccine-naïve immune adults in China. However, suboptimal protective responses may occur due to dose-series incompletion. We aim to investigate the immunogenicity of a 60 μg HB vaccine with a 0–2 month series among HBV vaccine-naïve immune adults aged 25–55 to assess potential alternative approaches for HB immunization. A two-center randomized controlled trial was carried out. Participants were randomly allocated to either the 20 μg (0–1–6 month) or the 60 μg (0–2 month) regimen. Blood samples were collected eight weeks after the final injection to measure the antibodies. A total of 583 adults (289 in the 20 μg regimen and 294 in the 60 μg regimen) were included. The seroprotection rates (SPRs) were 97.23% and 93.54% in the 20 μg and 60 μg regimens, respectively (*p* = 0.0261), and the geometric mean concentrations were 600.76 mIU/mL and 265.68 mIU/mL, respectively (*p* < 0.0001). The immunogenicity of the 60 μg regimen decreased significantly with age, particularly in adults aged 40 and older. The 60 μg regimen may be beneficial for adults under 40, especially those with poor compliance or in urgent need of immunization.

## 1. Introduction

Hepatitis B virus (HBV) is a significant challenge to public health, involving about 257 million people and resulting in 1.19 million deaths from HBV-related liver disease in 2015 worldwide [1]. The World Health Organization (WHO) has recommended vaccination as key to controlling HBV [2]. In China, the implementation of childhood vaccination in 1992 has significantly reduced HBV infection, from 9.7% in 1992 to 0.3% in 2014 in children aged 1–4. Despite this success, HBV prevalence remains high at 8–12% among adults aged 20 and older [3,4], prompting the Chinese Center for the Chinese Preventive Medicine Association (CPMA) and Disease Control and Prevention (CDC) to advocate for expanded adult immunization [5].

Currently, a regimen of three doses of 20 μg HBV vaccine is standard for adults in China [6]. However, vaccination coverage among adults is suboptimal, with studies indicating that up to 70% of adults aged ≥ 19 years who begin the three-dose series do not complete it [7], leading to insufficient immunological responses and shorter seroprotection durations [4]. Factors such as vaccine hesitancy/refusal [8], cost [9], and the logistical challenges of a multidose schedule contribute to these issues, often preventing adults from achieving full protective immunity within the optimal intervals [10]. Therefore, the ongoing quest is to develop the availability of HBV vaccines that require fewer doses and shorter intervals between doses accompanying sufficient seroprotection for adults, helping with the dose-series completion.

Recognizing these challenges, a new HBV vaccine containing 60 μg, produced via DNA recombination in yeast, was approved in China in 2010, primarily for non-responders or the immunocompromised [6,11]. In 2013, a study comparing the immunogenicity of the standard 20 μg three-dose regimen to a two-dose 60 μg regimen in healthy young adults aged 18–25 demonstrated that the 60 μg regimen was highly immunogenic and safe, suggesting a potentially simplified vaccination schedule that could improve compliance [12]. Given that vaccine responsiveness tends to decrease with age [13,14], the present study aimed to assess the immunogenicity of the two-dose 60 μg series versus the three-dose 20 μg regimen in adults without prior HBV vaccination between the ages of 25 and 55 years, exploring alternative approaches for hepatitis B immunization in this demographic.

## 2. Materials and Methods

### 2.1. Study Design

**Figure 1:**
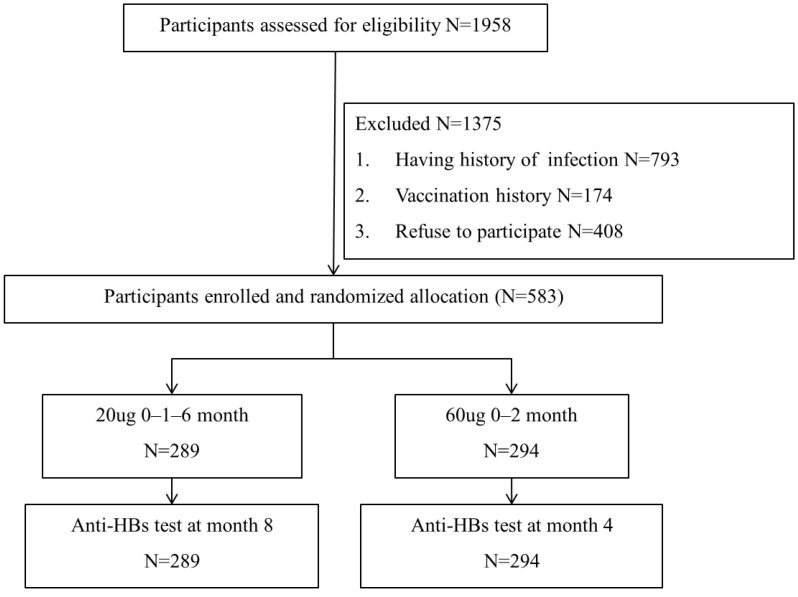
Flow chart of the participants through the study.

This prospective, randomized, two-center clinical trial was undertaken in Huaibei County, Anhui Province, and Xuanhua County, Hebei Province in 2013–2014. Participants were screened for antibodies against the HBV core antigen (anti-HBc) and surface antigens (anti-HBs and HBsAg) before vaccination. Individuals who tested negative for all three markers were randomly allocated to two groups using a computer-generated code. One group received the standard 20 μg hepatitis B vaccine on a 0–1–6-month schedule, while the other group received a 60 μg dose on a 0–2-month schedule. Blood samples for immunological analysis were taken 8 weeks post-final vaccination. Participants also completed a questionnaire detailing demographics, personal health data, and previous hepatitis B vaccinations. Figure 1 illustrates the study flow chart.

All participants provided written consent prior to the procedure. The study protocol received approval from both the medical ethics committees of Huai Bei People’s Hospital and The Second Affiliated Hospital of Hebei North University. The trial procedures complied with the Good Clinical Practice Guidelines and adhered to the ethical standards set forth in the Helsinki Declaration (Clinical trial registration: Identifier No. NCT06515938).

### 2.2. Study Population

Eligible participants were adults aged 25–55 years, negative for HBsAg, anti-HBs, and anti-HBc and without prior HBV or HAV vaccination. Exclusion criteria included a history of vaccine component allergies, autoimmune diseases, immunodeficiency, vaccinations received within the past month, acute illnesses within the past week, or fever (axillary temperature exceeding 38 °C) within the past three days.

### 2.3. Vaccines

The hepatitis B vaccines made by recombinant DNA techniques in yeast (Shenzhen Kangtai Biological Products Co., Ltd., Shenzhen, China) contained either 60 μg (Batch no: A201106003) or 20 μg (Batch no: B201202006) of HBsAg per 1.0 mL dose. The adjuvant systems used were aluminum hydroxide in sodium chloride and water for both vaccines. Vaccines were kept at 4 °C and the cold chain was maintained during transportation. Each vaccine was given intramuscularly in the deltoid muscle, alternating with each injection.

### 2.4. Serological Tests

Quantitative tests for anti-HBs, HBsAg, and anti-HBc were conducted pre-vaccination, and anti-HBs were tested 8 weeks post-final injection using the Architect i2000 system (Chemiluminescence Microparticle Immunoassay, Abbott, Chicago, IL, USA). The detection limits for anti-HBs ranged from 1 to 1000 mIU/mL.

### 2.5. Statistical Analyses

The primary outcome measured was the proportion of participants who had anti-HBs titers ≥ 10 mIU/mL (seroprotection rate, SPR) to HBV vaccination eight weeks after completion of vaccination.

The sample size was calculated according to the two rates comparison formula: 

*n*_1_ = *n*_2_ = 0.5[$\frac{\;z_{\alpha }+z_{\beta }}{{sin}^{-1}\sqrt{p_{1}}-{sin}^{-1}\sqrt{p_{2}}}$]^2^ (α = 0.05, β = 0.1), with the assumption that the SPR for the 20 μg regimen (*p*_1_) was 96% and was 90% for the 60 μg regimen (*p*_2_). The estimated sample size was 295 for each regimen.

Subsequently, 10 mIU/mL ≤ anti-HBs titers < 100 mIU/mL were considered a low response, 100 mIU/mL ≤ anti-HBs titers < 1000 mIU/mL were moderate response, and anti-HBs titers ≥ 1000 mIU/mL were hyper-response [15]; 95% confidence intervals (95%CI) and geometric mean concentrations (GMCs) were calculated using log-transformed antibody titers. Group comparisons were made using Pearson’s *χ*^2^ test, *χ*^2^ for trend, Fisher’s exact test for categorical data, and Student’s *t*-tests for continuous data. A two-tailed *p*-value < 0.05 was considered statistically significant. Analyses were performed using SPSS 18.0 software (SPSS Inc., Chicago, IL, USA).

## 3. Results

### 3.1. Demographics Analysis

Of the 1958 participants assessed for eligibility, 793 had history of hepatitis infection, 174 had history of prior HBV or HAV vaccination, and 408 refused to participate. In total, 583 participants were enrolled and randomized into two vaccination groups: 289 received the 20 μg regimen and 294 received the 60 μg regimen. Each participant completed the primary vaccination series and underwent serologic testing 8 weeks after the final injection. Demographic characteristics did not differ significantly between the two groups (Table 1).

### 3.2. Immunogenicity

The SPR at eight weeks post-final injection was markedly greater in the 20 μg group (97.23%) compared to the 60 μg group (93.54%; *p* = 0.0261). GMCs also differed significantly, with the 20 μg regimen achieving a GMC of 600.76 mIU/mL (95%CI: 495.27–728.65 mIU/mL) compared to 265.68 mIU/mL (95%CI: 214.97–328.39 mIU/mL) for the 60 μg regimen (*p* < 0.0001). Analysis of anti-HBs response categories indicated significant differences in the proportions of low and hyper responses between the two regimens (low response: 9.34% in the 20 μg group vs. 21.77% in the 60 μg group, *p* < 0.0001; hyper response: 40.14% in the 20 μg group vs. 21.09% in the 60 μg group, *p* < 0.0001). Moderate response rates did not differ significantly (47.75% vs. 50.68%; *p* = 0.4793).

As there was a high incidence of smoking in each group, further analysis on smoker subgroup was conducted and the results showed no significant difference between the participants with and without smoking either in the 20 μg group or the 60 μg group; the SPR were 97.37% vs. 97.18% in the 20 μg regimen (*p* = 0.6467) and were 93.81% vs. 93.40%; in the 60 μg regimen (*p* = 0.8922). GMCs also did not differ significantly, with the GMC of 679.87 mIU/mL (95%CI: 454.68–1016.58 mIU/mL) for participants with smoking compared to 574.79 mIU/mL (95%CI: 460.91–716.87 mIU/mL) for the non-smoking participants in the 20 μg group (*p* = 0.4152), and were 221.63 mIU/mL (95%CI: 150.36–326.69 mIU/mL) compared to 290.50 mIU/mL (95%CI: 225.47–374.28 mIU/mL) in the 60 μg group (*p* = 0.4444), respectively.

### 3.3. Immune Response of Adults in Different Age Groups

**Figure 2:**
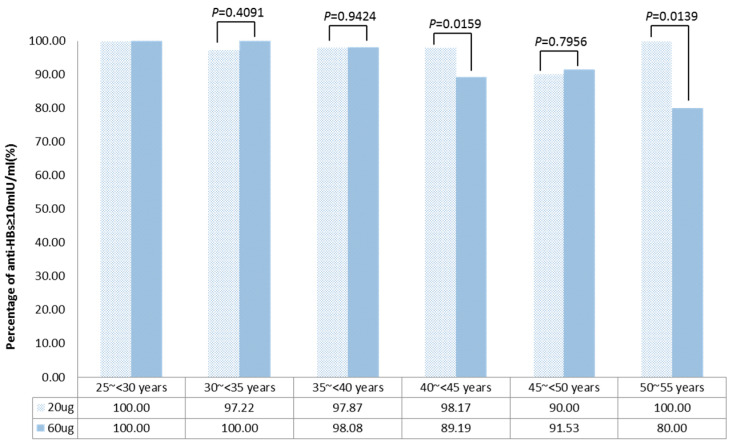
Age-specific percentages of antibody against hepatitis B virus surface antigen (anti-HBs) ≥ 10 mIU/mL for the 20 μg (0–1–6 month) and 60 μg (0–2 month) hepatitis B vaccination regimens.

**Figure 3:**
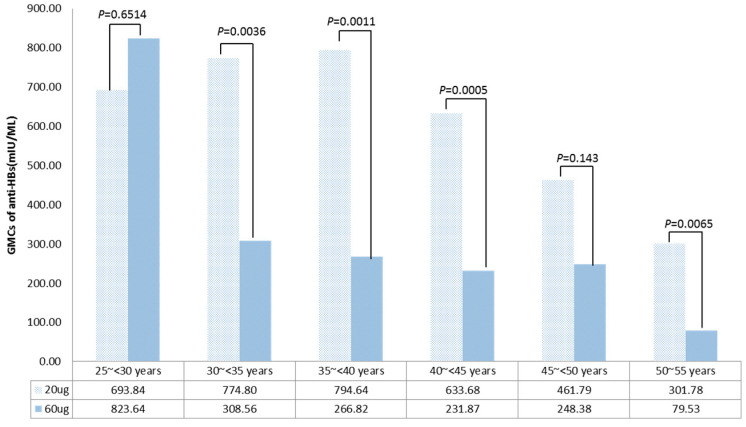
Age-specific geometric mean concentrations (GMCs) of antibody against hepatitis B virus surface antigen (anti-HBs) for the 20 μg (0–1–6 month) and 60 μg (0–2 month) hepatitis B vaccination regimens.

SPR and GMCs showed an age-related decline in the 60 μg group (*p* = 0.0019 and *p* = 0.0002, respectively), whereas the 20 μg group showed no significant age-related differences (Figure 2 and Figure 3). SPR varied significantly between the two vaccine groups among participants aged 40~<45 and 50~55 years (*p* = 0.0159 and *p* = 0.0139, respectively); other age groups showed comparable rates.

Age-specific GMCs did not differ significantly between the vaccine groups for participants aged 25~<30 and 45~<50 years (*p* = 0.6514 and *p* = 0.1430, respectively). However, GMCs were significantly higher in the standard 20 μg regimen for ages 30~<35, 35~<40, 40~<45, and 50~55 years (*p* = 0.0036, *p* = 0.0011, *p* = 0.0005, and *p* = 0.0065, respectively).

**Figure 4:**
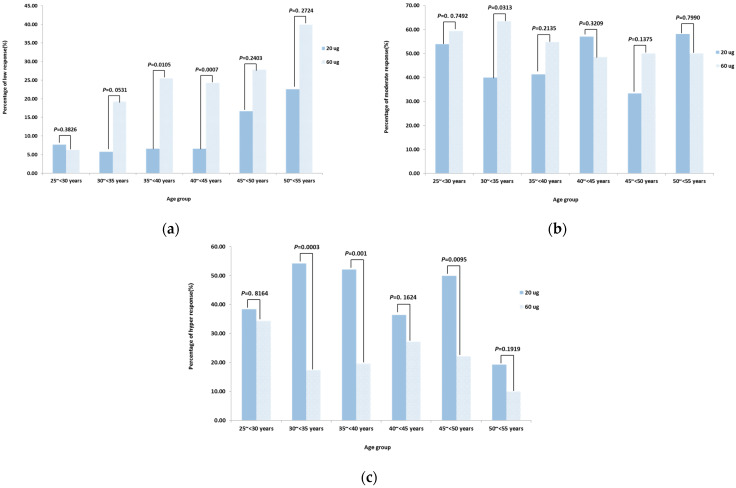
Percentage of category response for the 20 μg (0–1–6 months) and 60 μg (0–2 months) hepatitis B vaccination regimens stratified by age. (**a**) Percentage of low response (10 mIU/mL ≤ anti-HBs titers < 100 mIU/mL) for the 20 μg and 60 μg regimens stratified by age; (**b**) Percentage of moderate response (100 mIU/mL ≤ anti-HBs titers < 1000 mIU/mL) for the 20 μg and 60 μg regimens stratified by age; (**c**) Percentage of hyper response (anti-HBs titers ≥ 1000 mIU/mL) for the 20 μg and 60 μg regimens stratified by age.

Regarding anti-HBs category response (Figure 4), the distribution of anti-HBs levels did not differ significantly for the age groups 25~<30 and 50~55 years. In other age brackets, the standard 20 μg regimen achieved higher response rates, exhibiting a greater proportion of hyper responses (30~<35 years: *p* = 0.0003, 35~<40 years: *p* = 0.001, 40~<45 years: *p* = 0.1624, 45~<50 years: *p* = 0.0095) and a smaller proportion of low responses (30~<35 years: *p* = 0.0531, 35~<40 years: *p* = 0.0105, 40~<45 years: *p* = 0.0007, 45~<50 years: *p* = 0.2403).

## 4. Discussion

Considering the potential for accelerating vaccination schedules to improve compliance and provide rapid protection, this study compared the immunogenicity of a 60 μg hepatitis B vaccination requiring only a two-dose series with the standard 20 μg three-dose series in HBV vaccine-naïve immune adults between 25 and 55 years of age. The objective was the provision of a reference for assessing more appropriate immunization strategies for adults in China. Findings demonstrated that both vaccine regimens achieved protective rates of over 90%, with 97.23% for the 20 μg regimen and 93.54% for the 60 μg (0–2 month) regimen. Furthermore, the anti-HBs titers were higher in the 20 μg regimen at 8 weeks post-final injection, with GMCs of 600.76 mIU/mL compared to 265.68 mIU/mL for the 60 μg regimen.

Previous research has indicated that an anti-HBs titer of 10 mIU/mL or above in an adult two to three months after vaccination is likely to remain strong [16,17], conferring long-lasting immunity against HBV infection [18,19]. For example, Wu et al.’s study revealed an SPR of 92.3% five years post-vaccination using the 20 μg regimen [20], underscoring the importance of this regimen for achieving sustained immunogenicity. However, the rapid immunogenic response of the 2-dose 60 μg regimen may significantly benefit adults unable to complete the standard schedule due to compliance issues or those requiring immediate protection. This suggests that the 60 μg regimen could be a viable alternative, offering rapid and efficient immunization.

Supporting this, Ma Fubao et al.’s study found that a single dose of 60 μg of the HBV vaccine yielded an SPR of 85.7% in adults older than 16 years, with no adverse events reported, indicating acceptable immunogenicity and safety [21]. Similarly, Wang et al.’s study compared the immunogenicity of the standard 20 μg and higher-dose 60 μg regimens in young adults aged 18–25 years. The 60 μg regimen achieved a comparable SPR of 99.19% and GMCs of 1244.80 mIU/mL one month following the final injection [12]. Although the SPRs (97.23% and 93.54% for the two regimens) in this study were consistent with these findings, the observed GMCs were comparatively lower. This discrepancy is likely attributable to the older age demographic in this study, as immune response is known to decrease with age [22,23,24,25]. Furthermore, other individual factors such as drinking habits, smoking, obesity, gender, and concurrent diseases may also influence immunogenicity [13].

The impact of age on vaccine efficacy has been confirmed in this study, demonstrating that younger individuals experience an enhanced vaccine effect due to changes in cellular and humoral immune responses with age [13]. Thus, researchers have proposed adapting different hepatitis B vaccination strategies to different age demographics [25]. In order to provide a reference for the age-specific vaccination decision, the immunogenicity of different vaccination regimens was analyzed across age groups. Findings indicated that individuals younger than 40 years demonstrated comparable SPRs between the 60 μg (0–2 month) and 20 μg (0–1–6 month) regimens, with high SPRs (25~<30 years: 100% in each group; 30~<35 years: 97.22% vs. 100.00%; 35~<40 years: 97.87% vs. 98.08%, respectively) consistent with previously reported rates of 90% to 100% [26]. Notably, in adults aged 25~<30, both regimens achieved similar proportions of hyper response (34.38% in the 20 μg series vs. 38.46% in the 60 μg series) and a low proportion of low response (6.25% in the 20 μg series vs. 7.69% in the 60 μg series). However, other age groups displayed suboptimal responses, supporting the hypothesis that seroprotective antibody titers decline with age. The 60 μg (0–2 month) regimen could enhance vaccine compliance and coverage among adults younger than 40 years, offering a viable alternative for this age group.

For adults aged 40 years or older, the 60 μg regimen resulted in lower anti-HBs GMCs and a lower number of anti-HBs titers of at least 10 mIU/mL. Therefore, the 20 μg (0–1–6 month) regimen is preferable for ensuring adequate seroprotection in this older age group. Despite this, it is crucial to recognize that an initial peak anti-HBs level less than 10 mIU/mL is not necessarily indicative of an absence of immunity, as protection can persist for up to 30 years even when the anti-HBs level decreases below this threshold [27]. Approximately 10% of participants over 40 years of age in the 60 μg (0–2 month) regimen did not seroconvert, yet they were likely not unprotected due to the potential for an anamnestic response—a rapid, prominent increase in anti-HBs level triggered by HBsAg-specific memory B and T cells in the blood and lymph nodes, as well as the long-lived plasma cells and memory T cells in the bone marrow, which could persist in long-term regardless of anti-HBsAg and can be activated to expand rapidly upon HBV exposure or after receiving a booster dose. Several long-term follow-up studies revealed that the anti-HBs antibody declined with time after achieving initial vaccination immunity; those who become waning or undetectable of serum antibodies could still be protected by the vaccine-induced antibodies outlasting the well-preserved memory response when expose to HBV [28,29,30,31,32,33]. Based on current evidence, the three-dose 20 μg regimen is recommended for individuals aged 40 and older due to its superior immune response. However, the 60 μg (0–2 month) regimen remains a viable option for those at high risk of exposure or who may not complete the full three-dose schedule.

This study has several limitations. First, the long-term collection of blood samples is necessary to assess antibody persistence. Second, the immune response should also be monitored after each vaccination dose to detail trends. Third, vaccination history was self-reported via questionnaires, which may introduce recall bias.

## 5. Conclusions

In conclusion, this study revealed that the 60 μg two-dose regimen achieves a SPR exceeding 90% in HBV vaccine-naïve adults aged 25–55 despite lower GMCs than the standard three-dose 20 μg regimen. The findings support maintaining the standard three-dose 20 μg regimen as the optimal choice for adults due to its superior immune response and durability. However, for adults under 40 years of age, particularly those with poor compliance or in need of rapid protection, the 60 μg regimen requiring fewer injections presents a viable alternative, offering adequate immunogenicity within a shorter time frame.

## Figures and Tables

**Table 1:** Demographic information on participants.

	20 µg	60 µg	*p* Value
Subjects, no	289	294	
Age, Mean (SD)	40.3 (7.1)	39.5 (7.7)	0.1934
25~<30 years, N (%)	26 (9.0)	32 (10.9)	0.0116
30~<35 years, N (%)	36 (12.5)	52 (17.7)	
35~<40 years, N (%)	47 (16.3)	52 (17.7)	
40~<45 years, N (%)	109 (37.7)	74 (25.2)	
45~<50 years, N (%)	40 (13.8)	59 (20.1)	
50~55 years, N (%)	31 (10.7)	25 (8.5)	
Sex, N (%)			
Male	126 (43.6)	145 (49.3)	0.1661
Female	163 (56.4)	149 (50.7)	
BMI, Mean (SD)	23.8 (3.1)	23.9 (3.0)	0.4939
History of smoke, N (%)	76 (26.3)	97 (33.0)	0.0769
History of drink, N (%)	80 (27.7)	81 (27.6)	0.9719

## Data Availability

The de-identified data are available upon reasonable request from the corresponding author.
